# A Comparison of Three-Layer and Single-Layer Small Vascular Grafts Manufactured via the Roto-Evaporation Method

**DOI:** 10.3390/polym16101314

**Published:** 2024-05-08

**Authors:** Gualberto Antonio Zumbardo-Bacelis, Laura Peponi, Rossana Faride Vargas-Coronado, Eustolia Rodríguez-Velázquez, Manuel Alatorre-Meda, Pascale Chevallier, Francesco Copes, Diego Mantovani, Gustavo A. Abraham, Juan Valerio Cauich-Rodríguez

**Affiliations:** 1 Unidad de Materiales, Centro de Investigación Científica de Yucatán, Calle 43 #130 x 32 y 34, Colonia Chuburná de Hidalgo, Mérida C.P. 97205, Mexico; gualberto-antonio.zumbardo-bacelis.1@ulaval.ca (G.A.Z.-B.); ross@cicy.mx (R.F.V.-C.); 2 Department of Chemical Engineering, Laval University, Quebec, QC G1V 0A6, Canada; 3 Instituto de Ciencia y Tecnología de Polímeros (ICTP-CSIC), C/Juan de la Cierva 3, 28006 Madrid, Spain; 4 Facultad de Odontología, Universidad Autónoma de Baja California, Tijuana 22390, Mexico; eustolia.rodriguez@uabc.edu.mx; 5 Centro de Graduados e Investigación en Química-Grupo de Biomateriales y Nanomedicina, Tecnológico Nacional de México, Instituto Tecnológico de Tijuana, Tijuana 22510, Mexico; 6 Centro de Graduados e Investigación en Química-Grupo de Biomateriales y Nanomedicina, CONAHCYT-Tecnológico Nacional de México, Instituto Tecnológico de Tijuana, Tijuana 22510, Mexico; manuel.alatorre@tectijuana.edu.mx; 7 Laboratory for Biomaterials and Bioengineering (CRC-I), Department of Min-Met-Materials Engineering & CHU de Quebec Research Center, Laval University, Quebec, QC G1V0A6, Canada; pascale.chevallier@crchudequebec.ulaval.ca (P.C.);; 8 Research Institute for Materials Science and Technology, INTEMA (UNMdP-CONICET). Av. Colón 10850, Mar del Plata B7606BWV, Argentina

**Keywords:** roto-evaporation, vascular graft, burst pressure, compliance, mechanical properties, biomedical applications

## Abstract

This study used the roto-evaporation technique to engineer a 6 mm three-layer polyurethane vascular graft (TVG) that mimics the architecture of human coronary artery native vessels. Two segmented polyurethanes were synthesized using lysine (SPUUK) and ascorbic acid (SPUAA), and the resulting materials were used to create the intima and adventitia layers, respectively. In contrast, the media layer of the TVG was composed of a commercially available polyurethane, Pearlbond 703 EXP. For comparison purposes, single-layer vascular grafts (SVGs) from individual polyurethanes and a polyurethane blend (MVG) were made and tested similarly and evaluated according to the ISO 7198 standard. The TVG exhibited the highest circumferential tensile strength and longitudinal forces compared to single-layer vascular grafts of lower thicknesses made from the same polyurethanes. The TVG also showed higher suture and burst strength values than native vessels. The TVG withstood up to 2087 ± 139 mmHg and exhibited a compliance of 0.15 ± 0.1%/100 mmHg, while SPUUK SVGs showed a compliance of 5.21 ± 1.29%/100 mmHg, akin to coronary arteries but superior to the saphenous vein. An indirect cytocompatibility test using the MDA-MB-231 cell line showed 90 to 100% viability for all polyurethanes, surpassing the minimum 70% threshold needed for biomaterials deemed cytocompatibility. Despite the non-cytotoxic nature of the polyurethane extracts when grown directly on the surface, they displayed poor fibroblast adhesion, except for SPUUK. All vascular grafts showed hemolysis values under the permissible limit of 5% and longer coagulation times.

## 1. Introduction

Cardiovascular diseases (CVDs) remain the leading cause of death worldwide, even during the COVID-19 era. In fact, COVID-19 impacts the cardiovascular system in multiple ways, resulting in increased morbidity in patients with pre-existing cardiovascular conditions, as well as causing myocardial injury and dysfunction [1]. According to the World Health Organization [2], 33% of deaths worldwide can be attributed to CVDs. These diseases are typically characterized by the obstruction of blood flow through vessels and damage to the endothelial tissue, leading to a lack of nutrient supply [3]. The prevailing complications are coronary heart disease (CHD), peripheral artery disease (PAD), thrombosis, intimal hyperplasia, atherosclerosis and myocardial ischemia [4]. Common treatments for CVDs include the drastic modification of risk factors such as smoking cessation, blood pressure management, lipid control, weight loss, physical activity and dietary modifications [5]. However, in advanced cases of cardiovascular pathologies, a vascular surgery intervention (minimal or invasive) is required involving open surgical and endovascular procedures such as angioplasty, stenting, atherectomy and, in the worst scenario, the replacement or bypass of a damaged or occluded vessel [6]. Coronary artery bypass graft (CABG) surgery is the gold-standard procedure in patients with complex multivessel coronary artery disease [7]. The preferred conduits for vascular grafting are native veins and arteries, with saphenous vein (SV) being the most used autograft vessel [8]. Despite substantial advances in CABG surgery in the past decade, graft patency continues to be the ‘chink in the armor’ of this procedure. Moreover, autologous vessels are not always the best option due to the limited availability and poor quality of tissue from donors [4,9]. Failures related to saphenous vein grafts include mechanical mismatch, thrombosis, neointimal hyperplasia, stenosis and complete occlusion [10,11]. Synthetic vascular grafts are often considered the best alternative when autologous vessels are not available. The first successful vascular prosthesis replacement was performed by Voorhees in 1952 [12] using a porous fabric made from Vinyon “N” (PVC, polyvinylchloride). Ever since, the development of synthetic grafts has evolved significantly, and prostheses made from Dacron^®^ (PET, polyethylene terephthalate) [13] and expanded Teflon^®^ (ePTFE, polytetrafluoroethylene) [14] have been used as middle- and large-caliber grafts when autologous grafts are not available. These materials have been widely adopted due to their biocompatibility, durability and availability [15]. Not showing the same clinical progress as medium and large-diameter vascular grafts, small-diameter vascular grafts (≤6 mm) are seldom used due to their poor patency rates, which limit their application in coronary artery bypass graft surgery [3,16,17]. The optimal vascular substitute must be capable of mimicking native vessels; in other words, it should be mechanically strong, compliant, kink-resistant with good suture retention, non-toxic, non-immunogenic, non-thrombogenic and biocompatible [18].

Polyurethanes are versatile biomaterials that have found applications in various fields, including maxillofacial implants, non-adhesive barriers, controlled-release devices, and blood contact applications like catheters, aortic valves, coatings for pacemakers, and vascular grafts [19,20]. Among the different types of polyurethanes, segmented polyurethanes (SPU) have attracted significant attention due to their ability to adjust mechanical and biological properties by manipulating the chemical architecture using different hard and soft segments [21,22]. Polyurethanes can be used in their prepared/synthesized form [23], in a surface-functionalized form [24] and as scaffolds for soft [25] and hard tissue engineering [26], making them a versatile class of biomaterials. Taking advantage of the versatility of polyurethanes to match the properties required in the cardiovascular field, it is possible to design a small-caliber vascular graft, composed of a three-layer structure with different thicknesses and compositions comparable to the vascular wall of vessels. Artery and vein walls are organized into three layers, where the tunica intima is composed of endothelial cells covering the lumen; the tunica media is composed of elastin, collagen fibers and smooth muscle cells (SMCs) aligned circumferentially; and the tunica adventitia is formed of connective tissue and fibroblasts. The proportion of each tunica and the wall thickness is variable among small-, medium- and large-caliber veins and arteries [27]. With this structure in mind, several techniques have been proposed to develop vascular grafts, including electrospinning, bioprinting, 3D-printing [28,29], salt-leaching/the solvent casting method, films wrapped in a rod, molding [30,31,32] and even a combination of various techniques in a sequential manner with the same polymer composition [32,33].

Considering the low compliances exhibited by high-caliber vascular grafts made from expanded Teflon and knitted Dacron to date, there is no equivalent of these materials for their use in small vascular grafts. Therefore, the need for alternatives to PTFE and PET vascular grafts remains. In this regard, polyurethanes are an excellent alternative, as they have been used in several cardiovascular applications since 1960. Although polyurethane vascular grafts’ long-term stability and safety have been a significant concern due to uncontrolled biodegradation problems and biocompatibility issues without eliciting a chronic inflammatory response, they can be manufactured by various simple techniques and can be modified for improved performance.

In this work, we explored a technique based on the rotational solvent casting method (referred to as roto-evaporation) to form a three-layer vascular graft in a cylindrical mold, using different polyurethanes for each layer deposited sequentially. The inner layer (intima) was formed with a lysine-based segmented polyurethane (SPUUK), as it has been proven that alkaline amino acid-based polyurethanes are biocompatible with endothelial cells [30]. The media layer was deposited by using commercial polyurethane (Pearlbond 703 EXP). The outer layer (adventitia) was formed with an ascorbic acid-based segmented polyurethane (SPUAA), which has been proven to be non-cytotoxic to fibroblasts and osteoblasts [34]. We hypothesize that a semi-crystalline polyurethane can exhibit high compliances (similar or superior to common PTFE/PET grafts) despite having a high Young’s modulus by tailoring polyurethane composition and thickness. Therefore, the objectives of this paper are as follows: (i) to obtain three-layer small-caliber vascular grafts where each layer has a different composition; (ii) to compare the three-layer construct with small-caliber vascular grafts made from a single layer (only one segmented polyurethane) or a single layer obtained from physical blends of the three segmented polyurethanes; (iii) to assess their mechanical performance in simple tension and according to the ISO 7198 standard for cardiovascular grafts (longitudinal and circumferential strength, suture strength, burst strength and compliance); and (iv) to determine their cytocompatibility and their hemocompatibility (coagulation time and hemolysis).

## 2. Materials and Methods

### 2.1. Materials

Poly (ε-caprolactone) diol (PCL diol, Mn = 2000), 4,4′ (metylene-bis-cyclohexyl) diisocyanate (H_12_MDI), tin (II) 2-ethylhexanoate, L-lysine dihydrochloride (K) and L-ascorbic acid (AA) were purchased from Sigma-Aldrich (Milwaukee, USA). Dimethylformamide (DMF) from Sigma-Aldrich (Steinheim, Germany) and tetrahydrofuran (THF) from JT Baker (Phillipsburg, USA) were used as received. Thermoplastic Polyurethane Pearlbond 703 EXP was obtained from Lubrizol^®^ and used as a model polyurethane for the core of the vascular graft. This commercial thermoplastic polyurethane is a hydrolytic-resistant aromatic-based polymer (toluene diisocyanate) with polycaprolactone diol as the soft segment, as demonstrated by FTIR, Raman and ^1^H NMR. It has a T_g_ of −36 °C (measured by DMA), a melting range between 40 °C and 84 °C (measured by DSC) and a decomposition temperature at 389 °C (measured by TGA), and it is considered as EU-Food-compliant (10/2011 regulation).

### 2.2. Synthesis of Segmented Polyurethanes

**Scheme 1:**
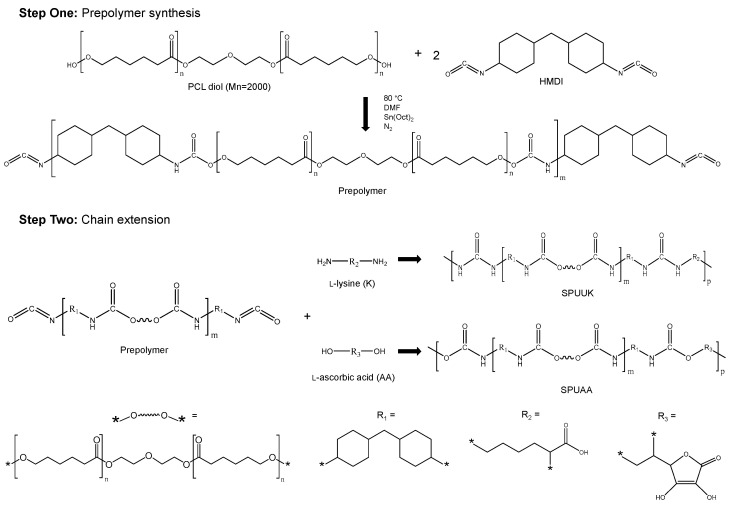
Synthesis route for SPUUK and SPUAA.

Segmented polyurethanes and urea were synthesized with a molar ratio of 1:2:1 (PCL: H_12_MDI: K or AA), as previously reported [34]. During the pre-polymer formation, PCL diol was dissolved in DMF and then reacted with a molar excess of H_12_MDI added dropwise in the presence of tin (II) 2-ethylhexanoate as a catalyst. The reaction was conducted under a dry nitrogen atmosphere and stirred for 4 h at 80 °C. For the chain extension reaction, either K or AA, previously dissolved in DMF, was added to the pre-polymer and left to react for another 2 h under constant stirring. To stop the reaction, the DMF dissolved polymer was precipitated in distilled water and stirred overnight. The polymer was thoroughly washed to eliminate the residual monomers and dried at 65 °C in vacuum. The obtained polymers were named as SPUUK for lysine-based polyurea urethane and SPUAA for ascorbic acid-based polyurethane. The chemical structure proposed for the SPUUK and SPUAA is shown in Scheme 1. Polyurethane synthesis was confirmed by FT-IR and Raman spectroscopy.

### 2.3. Physicochemical Characterization

Fourier transform infrared (FT-IR) spectra were recorded with a Nicolet 8700 spectrometer (Thermo Scientific, Madison, WI, USA) using attenuated total reflectance (ATR). A ZnSe crystal was pressed against the samples and each spectrum was collected between 4000 and 650 cm^−1^, with 100 scans and a resolution of 4 cm^−1^. Raman spectra were acquired using a Renishaw inVia Reflex Raman spectrometer (Wotton-under-Edge, Gloucestershire, UK) in the spectral range of 3200–200 cm^−1^. A 633 nm laser was used as the excitation radiation source using 50% of the total power, a 50× objective lens and 10 ms of exposure.

### 2.4. Rationale/Justification of the Roto-Evaporation Technique for Manufacturing Vascular Grafts

Most standard techniques for manufacturing experimental vascular grafts use electrospinning or wrapping onto a mandrel. The first provides suitable wall thickness with long processing times, while in the second, delamination occurs. Roto-evaporation offers a good alternative incorporating a different composition and thickness layer without blending the other biodegradable polyurethanes. The addition of the second layer did not dissolve the first layer as they have various polymer concentrations and, therefore, different densities and viscosities. In addition, the fast evaporation of THF did not allow the penetration and dissolution of a larger polyurethane block (typically, dissolution time for film forming was 24 h at 25 °C). Because of this, the different layers do not mix, demonstrating the adequacy of the technique for manufacturing vascular grafts where the intima layer can be thin, whereas the media layer can be thicker. However, this method is limited as it does not provide polymer alignment in other directions other than circumferential directions.

**Figure 1:**
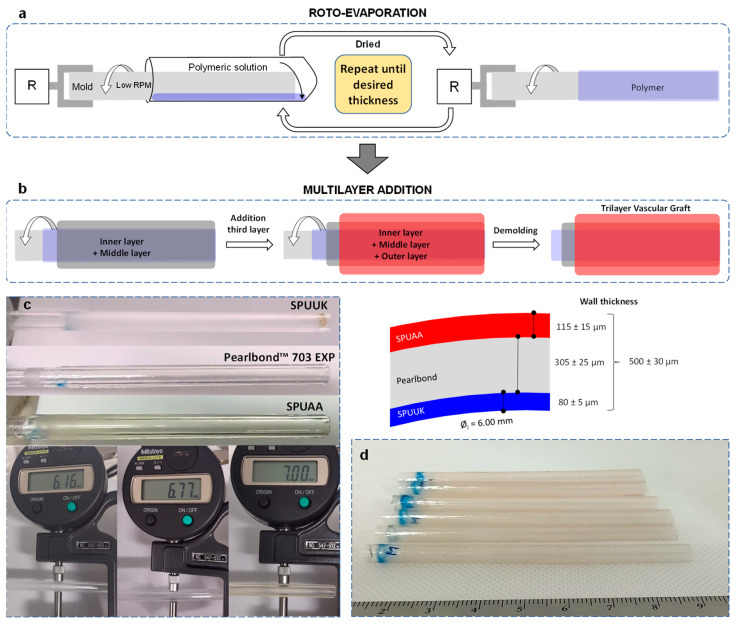
(**a**) Process flowchart of “Roto-evaporation” to fabricate vascular grafts, (**b**) multilayer addition to obtain multilayer vascular grafts, (**c**) digital images measuring the diameter of different layers added and (**d**) three-layer vascular grafts (TVGs) demolded.

To obtain dense tubular structures, a 6 mm diameter glass rod was immersed for 5–10 s in a low viscosity polyurethane solution (1 g polymer/30 mL THF). Then, the glass rod was placed horizontally on a Caframo^®^ overhead stirrer rotating at 60 rpm within a glass test tube to slow the rate of evaporation, as depicted in Figure 1a,b. The diameter of the glass rod plus the coating of the polymer was measured with a micrometer Mitutoyo model No. 547-512. This procedure was repeated until coronary artery thickness was achieved (see Figure 1c); for the single-layer grafts, the wall thickness was 0.132 ± 0.01 mm, according to the mean thickness of the intimal tunica in the coronary artery [35].

### 2.5. Fabrication of Polyurethane Three-Layer Vascular Graft

Based on high-frequency ultrasound imaging, the coronary artery has a wall thickness of 0.5 ± 0.1 mm, of which 16 ± 2% corresponds to the tunica intima, 62 ± 10% corresponds to the tunica media and 21 ± 5% corresponds to the tunica adventitia [35,36,37,38]. Therefore, to obtain the three different polymer solutions of TVG based on SPUUK, Pearlbond 703 EXP and SPUAA were prepared and deposited in a sequential manner. The inner layer of the vascular graft was made using SPUUK until reaching a diameter of 6.16 ± 0.01 mm (*w*_SPUUK_ = 0.08 mm for a 6 mm glass rod diameter), while the middle layer was made using Pearlbond 703 EXP, and the diameter of the SPUUK + Pearlbond was Ø_SPUUK+Pearlbond_ = 6.77 ± 0.05 mm (*w*_SPUUK+Pearlbond_ = 0.385 mm). Finally, the outer layer was made from SPUAA, and the final diameter was Ø_SPUUK+Pearlbond+SPUAA_ = 7.00 ± 0.03 mm (*w*_(SPUUK+Pearlbond+SPUAA)_ = 0.500 mm). All roto-evaporated tubular grafts in this study had an internal diameter of 6.00 mm. A summary of the process is showed in Figure 1. To assess the contribution of each individual polyurethane, tubular structures were fabricated with only SPUUK, Pearlbond 703 EXP and SPUAA, referred to as SVG. In addition, a physical blend of the three types of polyurethanes (MVG) was prepared by dissolving each polyurethane in THF (1:1:1 weight ratio of SPUUK/Pearlbond 703 EXP/SPUAA).

### 2.6. Mechanical Properties of Polyurethane Films and Vascular Grafts

#### 2.6.1. Tensile Properties of Polyurethane Films

The films used for the tensile tests were prepared by means of solvent evaporation. For this, each polyurethane was dissolved in tetrahydrofuran assisted by magnetic stirring for 24 h. Then, the polymeric solution was poured onto a 120 × 120 mm^2^ non-stick mold and left for 24 h to evaporate the solvent. Mechanical tensile tests were carried out in a universal testing machine MiniShimadzu AGS-X (Kyoto, Japan) equipped with a 100 N load cell with a resolution of 0.01 N and operated at a crosshead speed of 50 mm/min according to ASTM-D882 [39] at room temperature. The samples were dumbbell specimens 25 mm in length, 5 mm in width in the narrow section and 100 to 150 μm in thickness. The Young’s modulus (E) was calculated from the initial slope of the strain–stress curves in the strain range of 10%, the ultimate tensile strength ($\sigma_{max}$) and strain to failure ($\varepsilon_{max}$) are reported for at least 10 specimens.

#### 2.6.2. Tensile Properties of Vascular Grafts

**Figure 2:**
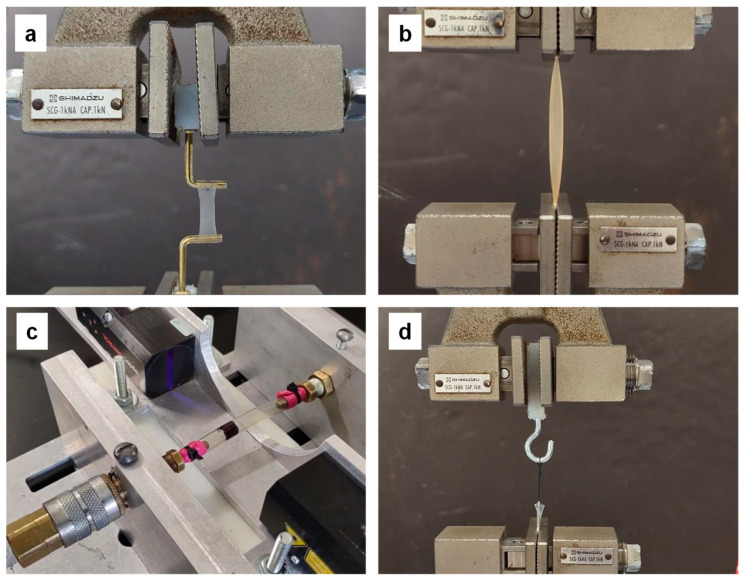
The fixtures for different mechanical tests of the tubular vascular grafts: (**a**) circumferential tensile strength; (**b**) longitudinal tensile strength; (**c**) burst strength in the custom-made equipment; and (**d**) suture retention strength.

The circumferential and longitudinal tensile properties of tubular structures (vascular grafts) were determined according to Sections 8.3.1 and 8.3.2, respectively, of the ISO 7198 standard [40] for cardiovascular implants and tubular vascular prostheses (ISO 7198:1998). Figure 2a shows the fixture used for measuring the circumferential tensile strength of 7 mm long specimens ($L_{C}$ = 7 mm) loaded along the tube radial direction and using two metallic hooks (diameter ($d_{hook}$) = 2.4 mm) until the failure of at least 10 samples. For the circumferential tensile test, the ISO 7198 standard recommends reporting the maximum force reached ($F_{C}^{max}$) divided by 2 times the length of the specimen ($F_{C}^{max}/2L_{C}$). This was referred to as the “circumferential tensile strength”, while the maximum displacement during the circumferential test was labeled as $\delta_{C}^{max}$. For longitudinal tensile strength (see Figure 2b), 8 tubular specimens with a nominal length of 50 mm ($L_{L}$ = 50 mm) were used and loaded along the tube axial direction (lengthwise) until failure. Both tests were carried out in a universal machine, MiniShimadzu AGS-X (Kyoto, Japan), equipped with a 1 kN load cell and operated at a crosshead speed of 50 mm/min according to ISO 7198 standard. The tensile force (F) as a function of crosshead speed displacement (δ) was recorded and the parameters reported were the maximum force or “longitudinal tensile strength”, labeled as $F_{L}^{max}$, and the “maximum displacement” of the crosshead machine upon failure during the test, labeled as $\delta_{L}^{max}$.

#### 2.6.3. Burst Strength

The burst strength test was conducted using a custom-made piece of equipment designed and manufactured for this type of test [41], as shown in Figure 2c. An 80 mm long vascular graft was pressurized (0.3 psi/s, ~15.5 mmHg/s, for all tests herein) using compressed air. By means of an optoCONTROL 1200-30 micrometer from Micro-Epsilon, Ortenburg, Germany, the external diameter ($D_{0}$) displacement was measured. The burst strength was calculated from the maximum pressure average of 8 specimens when these ruptured. Furthermore, the theoretical burst pressure was also calculated with the simplified Lame’s equation (Equation (1)):

(1) $$P_{i}=\sigma_{\theta }\frac{\left(D_{ex}^{2}-D_{in}^{2}\right)}{\left(D_{ex}^{2}+D_{in}^{2}\right)}\left[MPa\right]$$

where $\sigma_{\theta }$, $P_{i}$, $D_{in}$ and $D_{ex}$ are the hoop stress (≈Tensile stress, MPa), internal pressure (MPa), failure inner diameter (mm) and failure outer diameter (mm) of the theoretical tube, respectively [42]. The hoop stress at failure was calculated using Equation (2), where $F_{C}^{max}$ is the maximal load at failure (N), $L_{C}$ is the initial length (mm) and t is the thickness (mm) of the ring specimen.

(2) $$\sigma_{\theta }^{max}=\frac{F_{C}^{max}}{2L_{C}t}\left[MPa\right]$$

To calculate the failure inner diameter ($D_{in}$) in the specimens during the circumferential test, Equation (3) was used where $C_{fail}$ is the real internal circumference of the ring specimen at failure, $d_{hook}$ is the diameter of the hooks and $\delta_{c}^{max}$ is the distance of displacement between the hooks [43].

(3) $$D_{in}^{fail}=\frac{C_{in}^{fail}}{\pi }=\frac{d_{hook}\left(\pi +2\right)+2\delta_{c}^{max}}{\pi }\left[mm\right]$$

The failure external diameter was calculated with Equation (4) assuming the incompressibility of the wall thickness of the tube, where t is the wall thickness.

(4) $$D_{ex}=D_{in}+2t\;[mm]$$

Circumferential strain ($∆D/D_{0}$) was calculated as the change in the external diameter divided by the initial external diameter.

#### 2.6.4. Compliance

Circumferential compliance (C) was calculated from the experimental data at three different pressure ranges—low pressure (50 to 90 mmHg), medium pressure (80 to 120 mmHg) and high pressure (110 to 150 mmHg). Compliance was computed from recordings of inner pressure, $P_{i}$, and a computed inner diameter, $D_{P}^{in}$, through Equation (5) [42]:

(5) $$C=\frac{\frac{D_{H}^{in}-D_{L}^{in}}{D_{L}^{in}}}{P_{H}-P_{L}}\times 10^{4}\;[\%/100\;mmHg]$$

where $D_{H}^{in}$ is the internal diameter corresponding to the higher-pressure value and $D_{L}^{in}$ is the internal diameter corresponding to the lower-pressure value. The internal diameter was calculated using Equation (3). Pressure and diameter values were used to calculate a dimensional stiffness index, β, by means of Equation (6) [42,44]:

(6) $$\beta =\frac{\ln\left(\frac{P_{H}}{P_{L}}\right)}{\left[\frac{D_{H}^{ex}-D_{L}^{ex}}{D_{L}^{ex}}\right]}$$

#### 2.6.5. Suture Retention Strength

According to the ISO 7198 standard (Section 8.8), the suture retention strength test determines the force necessary to pull a suture from the prosthesis or cause the wall of the prosthesis to fail (ISO 7198:1998). The suture retention strength was measured using a 3-0 Nylon suture (Johnson & Johnson), as depicted in Figure 2d. For this, a tubular specimen was cut normal to the long axis and the suture was stitched at 2 mm from the end of the vascular graft forming a loop. Then, sutured specimens were placed on the universal testing machine MiniShimadzu AGS-X (Kyoto, Japan) equipped with a 1 kN load cell operated at a crosshead speed of 50 mm/min.

### 2.7. Scanning Electronic Microscopy (SEM)

The morphology and imaging of the grafts before and after testing were obtained using a scanning electron microscope, JEOL, JMS 6360LV (Akishima, Tokyo, Japan), operating in low-vacuum mode. The microscope was operated at 20 kV. All samples were coated with gold in a Desk II Sputter Coater (Moorestown, NJ, USA) at 60 s and 40 mA.

### 2.8. Biological Characterization of Pristine Polyurethanes

#### 2.8.1. Screening Test for Cytocompatibility

Cytocompatibility screening tests were carried out by means of elution (indirect) assays employing MDA-MB-231 as testing cells (breast cancer cells) and CCK-8 and crystal violet as testing probes. MDA-MB-231 (purchased from the American Type Culture Collection) cells were chosen for this experiment because of their highly proliferative behavior. Working with robust and highly proliferative cells in screening cytocompatibility/cytotoxicity tests compensates for parameters/conditions that might lead to false results (either positive or negative), which might be observed when employing cells with regular (moderate) growth. Examples of such parameters/conditions include the presence of reductive chemicals and metal ions in the extract or culture media, even at trace concentrations (which alter the reduction and color conversion of colorimetric testing probes, CCK-8 in our case) [45,46] and minute gradients in topography and water contact angle of cell substrates (e.g., tissue culture plates, which are known to impact the adhesion and proliferation of viable cells) [47]. Parameters like these or their combinations have been found to cause interferences when working with colorimetric probes, leading to either negative or positive false results from cytocompatibility tests when caution is not observed [48]. These tests were carried out according to the ISO 10993−5:2009 elution method with subtle variations [45]. Briefly, 20 mg of each polyurethane was cut into small pieces and immersed in 2 mL of culture medium and incubated under standard culture conditions for 48 h to produce extracts. MDA-MB-231 cells were seeded in 96-well plates at a density of 1000 cells/well with 100 μL of the cell culture medium and incubated for 24 h. Then, the culture medium was withdrawn and 100 μL of the extracts were added to the wells and incubated for 24 and 48 h; the cell culture medium was used as a control. Afterwards, the culture medium was replaced with 100 μL of fresh culture medium administered 10 μL of CCK-8 cell proliferation reagent and incubated for 4 h. Optical absorbance was measured afterwards at 450 nm using an ELISA microplate reader (BIO-RAD model iMark, USA). Cytocompatibility after the exposure of cells to sample extracts, represented as the percentage of cell viability, was calculated using Equation (7):

(7) $$Cell\;viability\left(\%\right)=\frac{A_{Ext}}{A_{Ctrl}}\times 100$$

where $A_{Ext}$ is the absorbance of cells after exposure to extracts and $A_{Ctrl}$ is the absorbance of the cell control, non-exposed cells (100% viability). The results were the average of six independent experiments for each time. The cytocompatibility of the prepared samples was also determined via the well-known crystal violet staining method [49]. To this end, the MDA-MB-231 cells were seeded in 96-well plates (100 µL, 15,000 cells/well) and grown for 24 h under standard culture conditions. Afterwards, the samples were internalized (100 µL) and incubated for the chosen period (24 h and 48 h). Then, the culture medium was discarded and the cells were attached to the plate in the presence of 10 µL of a glutaraldehyde solution (11 *w/v*% in water). The solution was discarded and cells were washed two times with Milli-Q water. The cells were then shaken at room temperature (300 rpm, 15 min) in the presence of 100 µL of a crystal violet solution (0.1 *w/v*% in 200 mM orthophosphoric acid, 200 mM formic acid and 200 mM MES, pH 6). The solution was discarded and the cells were again washed twice with Milli-Q water. Once washed, the cells were incubated at room temperature overnight for drying. Once dried, the cells were shaken at room temperature (300 rpm, 15 min) in the presence of 100 µL of acetic acid (10 *w/w*% in water). Immediately after this, the absorbance of the resulting solution was measured at 595 nm. The percentage of cell viability was quantified using Equation (7). The results were the average of six independent experiments. Sterile conditions were preserved up until cell fixation with glutaraldehyde.

#### 2.8.2. Cell Adhesion and Proliferation

A live/dead assay was performed to investigate the viability of cells in direct contact with the samples. For this, flat samples of 5 × 5 mm^2^ of each polyurethane were cut and placed in a 96-well plate, where fibroblast L–929 cells (1.5 × 10^4^ cells/well, 100 μL) were seeded and incubated under culture conditions for 24 h. Then, the culture medium was withdrawn, 3 μL of a solution 98:1:1 of PBS/calcein-AM/propidium iodide aqueous solution was pipetted on top of each sample and the samples were incubated for 10 min at room temperature and dark. Experiments were carried out in duplicates for each kind of sample. The samples were mounted on coverslips and observed using a LEICA SP8 confocal microscope (LEICA Microsystems Heidelberg GmbH) operating at wavelengths of 488/495–545 and 552/590–700 nm for the detection of calcein AM (green) and propidium iodide (red), respectively.

Both cell lines were cultured in a glucose-rich DMEM culture medium containing phenol red, supplemented with 10% FBS, 1% pen-strep, 1% non-essential amino acids (NEAA) and 1% sodium pyruvate. The materials were acquired from Sigma-Aldrich (DMEM, pen-strep, NEAA and sodium pyruvate) and Biotecfron (FBS). Throughout the experiments, sterile Milli-Q water was utilized. All samples were sterilized to ensure sterility by immersing them in 70% ethanol for 10 min before characterization. The humidified atmosphere was kept at 5% CO_2_ and the temperature was maintained at 37 °C.

#### 2.8.3. Clot Formation

To evaluate clotting times, the free hemoglobin method was used on 15 mm-diameter samples of the different polyurethanes [50]. First, samples were cleaned in an ultrasonic bath with 70% alcohol (*v*/*v*) for 10 min and then sterilized with UV light for 30 min on each side. For each sample, 100 μL of citrated blood was placed onto the surfaces and 20 μL of CaCl_2_ was added immediately. Finally, samples were incubated at 37 °C for 5, 10, 20, 30 and 40 min to study the kinetics of clotting. After these incubation times, 2 mL of deionized water was added and blood cells not entrapped in a thrombus were hemolyzed. One minute later, 100 µL aliquots of the solution containing the hemoglobin were transferred to a 96-well plate to perform absorbance reading. Free hemoglobin molecules in water were measured by monitoring the absorbance at 545 nm using a spectrophotometer ELISA reader (BioRad mod. 450 Mississauga, ON, Canada). The higher detected quantity of hemoglobin is correlated with the low level of clot formation in the presence of the samples. The test was performed in triplicate with 6 samples per condition each time. Blood from a different donor was used for each experiment.

#### 2.8.4. Hemolysis

For an early assessment of the possible hemotoxic effects of the different polymers used, a hemolysis test was conducted based on the ASTM Standard F756-17 (Standard Practice for Assessment of Hemolytic Properties of Materials) [51]. We collected whole human blood from healthy donors using citrate-containing blood collection tubes for this experiment. We placed three samples for each condition in a 15 mL tube and added 10 mL of sterile PBS 1X to each tube. PBS 1X was used as a negative control and deionized H_2_O was used as a positive control. Samples and controls were incubated at 37 °C for 30 min. In the meantime, the collected blood was diluted in PBS 1X to a final ratio of 4:5 (4 parts of citrated blood and 5 parts of PBS 1X). After the incubation, 200 μL of diluted blood were added in each tube and carefully mixed by inverting each tube. After that, samples and controls were incubated at 37 °C for 1 h. All tubes were carefully mixed via inversion after 30 min of incubation. At the end of the incubation, the tubes containing the samples and the controls underwent a centrifugation step at 800 g for 5 min. The supernatant was collected and 100 μL aliquots were placed in a 96-well plate. The absorbance (OD) at a wavelength of 545 nm was recorded and the hemolysis percentage was calculated according to Equation (8).

(8) $$Hemolysis\left(\%\right)=\frac{A_{S}-A_{N}}{A_{P}-A_{N}}\times 100$$

where $A_{S}$ represents the sample absorption value, $A_{N}$ is the negative control absorption value and $A_{P}$ is the positive control absorption value.

### 2.9. Statistical Analysis

Data were expressed as means ± the standard deviations of means. Significant differences were determined by running a one-way ANOVA followed by Tukey’s post hoc method to test all possible pairwise comparisons and determine where the differences lay. A value of *p*-value < 0.05 was considered statistically significant.

## 3. Results and Discussion

Segmented polyurethane-ureas were synthesized with a molar ratio of 1:2:1 (PCL: H_12_MDI: K or AA), leading to a rigid content of approximately 38 wt%. During prepolymer formation and by using an FDA-approved catalyst (a commonly used catalyst for the ring opening polymerization of L-lactide and glycolide used in the synthesis of PLA or PGA), the urethane group is formed, but due to the H_12_MDI excess used in the first step, prepolymers and free isocyanates are present. Then, the isocyanate-terminated soft segment was extended into a polymer by adding either lysine or ascorbic acid. In the case of lysine, the reaction of isocyanates with amines introduces a urea group. In contrast, an amide is also formed in the second reaction between isocyanates and carboxylic acids. Furthermore, chemical reactions between either the urethane or urea groups and isocyanates might form allophanates and Biuret products.

### 3.1. Physicochemical Characterization

**Figure 3:**
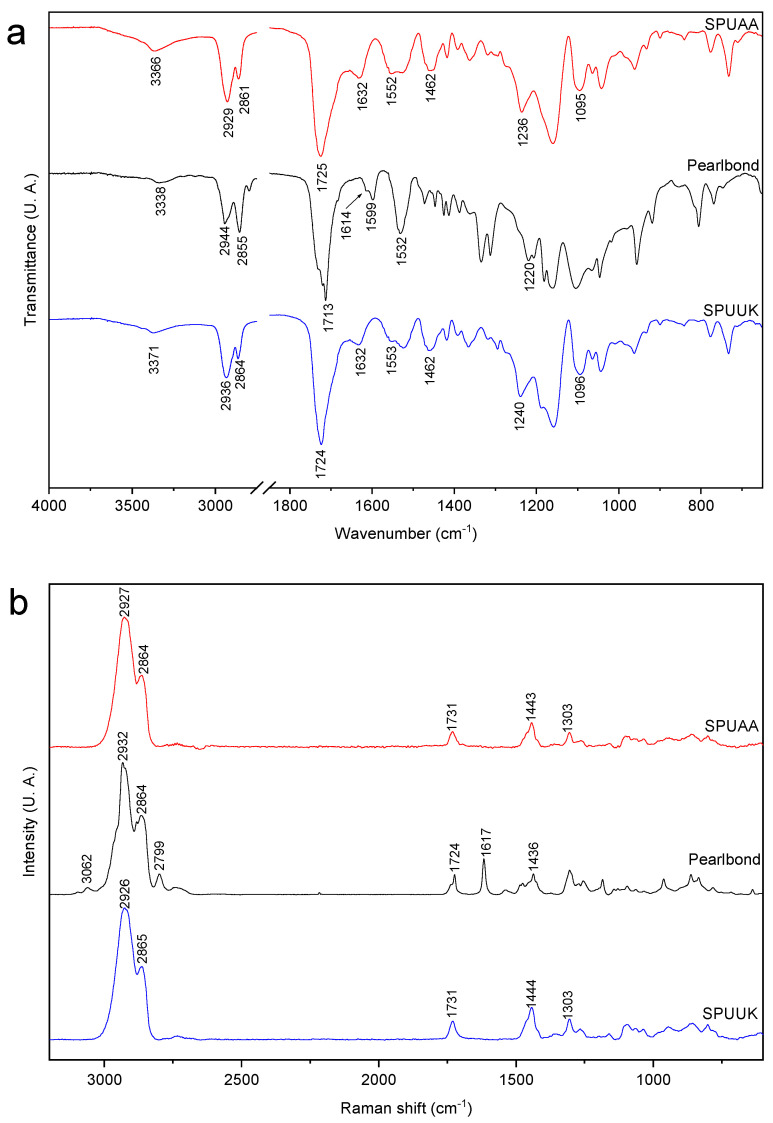
FT-IR spectra (**a**) and Raman spectra (**b**) of SPUUK, Pearlbond 703 EXP and SPUAA.

FT-IR spectra of SPUUK, Pearlbond 703 EXP and SPUAA are shown in Figure 3a, demonstrating the suggested structures proposed in Scheme 1. FT-IR spectra of the SPUUK and SPUAA show the ‘amide bands’, free –NH of urethane bond absorbance at 3300–3400 and amide II band (C–N stretching + N–H bending) at 1552 cm^−1^ [52]. The peaks observed at ~2930 and ~2862 cm^−1^ are associated with the asymmetric and symmetric –CH_2_ groups. The peak at ~1725 cm^−1^ is assigned to the stretching vibration of C=O ester groups. The peaks located at ~3365, 1725, 1462 and 1095 cm^−1^ correspond to the –NH, –C=O, –CH_2_ and –C–O absorptions and confirm the presence of NHCOO groups in the synthesized polyurethanes [53]. In FT-IR spectrum of Pearlbond 703 EXP, the band at 3338 cm^−1^ is caused by the stretching vibration of the N–H bond of the urethane. The bands at 2944 and 2855 cm^−1^ can be ascribed to asymmetric and symmetric –CH_2_ groups. The intense band at 1713 cm^−1^ represents stretching vibrations of the carbonyl groups and may be associated with the ester and to urethane carbonyls. The band at 1599 cm^−1^ can be associated at –C=C– stretching vibration of the aromatic ring in the diisocyanate [54]. The Raman spectra of the polyurethanes is shown in Figure 3b. The Raman bands at ~2930 and ~2862 cm^−1^ is associated with asymmetric and symmetric CH stretching vibrations of the –CH_2_ groups [55]. C=O stretching vibrations of urethane and ester groups can be observed at 1731 cm^−1^ in the SPUUK and SPUAA and 1724 cm^−1^ in the Pearlbond 703 EXP. The intense band at 1617 cm^−1^ in the Pearlbond 703 EXP spectrum is associated with –C=C– stretching vibrations and the non-intense band at 3061 cm^−1^ is associated with the =C– H of aromatic ring [56].

The absence of absorption peaks at 2260 cm^−1^ proved the absence of residual isocyanate. Furthermore, DMF and tin (II) 2-ethylhexanoate were not detected, either by FTIR/Raman or during biological studies, possibly because of their low concentration and thorough washing during polymer synthesis. However, it is recommended that ^1^H NMR be used for better quantification. Therefore, the safety of these polyurethanes is guaranteed. From a chemical point of view, few differences were detected between SPUUK and SPUAA, considering that they share the same soft segment content. In addition, as demonstrated later, they exhibited different mechanical behavior and biological properties.

### 3.2. Tensile Properties of Films and Tubes

**Figure 4:**
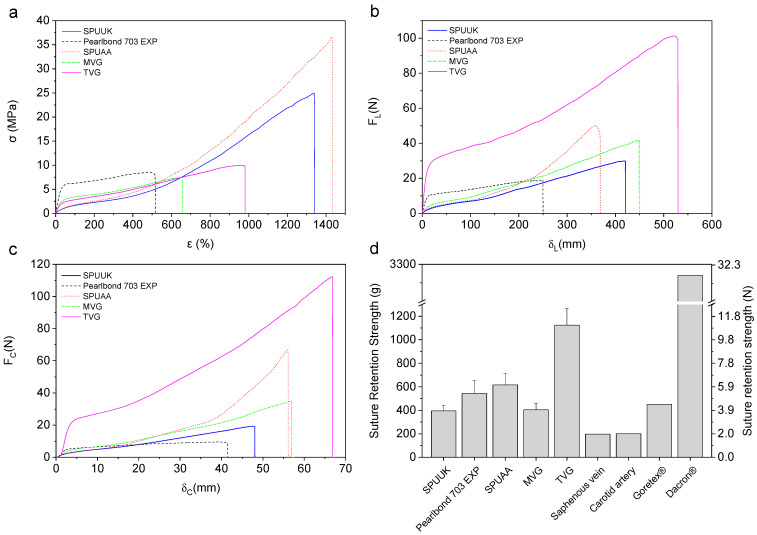
Representative curves of the mechanical behavior of films, tubes, and rings under the tensile loading of SPUUK, Pearlbond 703 EXP, SPUAA, polymer blends (MVGs) and three-layer vascular graft (TVGs) for (**a**) films under uniaxial tensile loading, (**b**) tubes under longitudinal load, (**c**) rings under circumferential load and (**d**) the suture retention strength test.

Uniaxial tensile testing representative curves of pristine SPUUK, Pearlbond 703 EXP, SPUAA and films made of a polyurethane blend are shown in Figure 4a. It was observed that under tension, SPUUK and SPUAA had a similar mechanical behavior, i.e., a deformation higher than 1000%, because they share 95% of their chemical structure. In contrast, Pearlbond 703 EXP showed lower deformation and ultimate tensile strength but a higher Young’s modulus than SPUUK and SPUAA. Therefore, the physical mixture or polyurethane blend showed intermediate values of the ultimate tensile strength (UTS) and deformation between SPUUK/SPUAA and Pearlbond 703 EXP. A rectangular strip cut from the TVG was also loaded under tension; only samples that broke in the middle section were considered. Assuming that there is no significant difference between a dumbbell and a rectangular sample in terms of the Young’s modulus but that failure properties may be affected, TVG tensile properties were lower in terms of tensile strength and deformation than SPUUK and SPUAA. Figure 4b displays the single-layer grafts assessed under longitudinal deformation, revealing a consistent trend, SPUAA exhibited the highest longitudinal load (49.8 ± 8.3 N), while the lowest was observed for Pearlbond 703 EXP (18.1 ± 4.3 N), being lower compared to three-layer vascular grafts, TVGs (101.3 ± 4.0 N).

In agreement with previous results (see Figure 4c), SPUUA showed the maximum circumferential strength (5.00 ± 0.61 N/mm), followed by SPUUK (1.49 ± 0.39 N/mm), while the lowest circumferential force was achieved by the polyurethane Pearlbond 703 EXP (0.87 ± 0.11 N/mm), which seems to be composed of MDI as the isocyanate used for its synthesis. Polymer blending led to an intermediate value between SPUAA and Pearlbond 703 EXP, even when they were at the same weight percentage. However, the TVG exhibited the highest circumferential tensile strength (7.17 ± 0.37 N/mm), suggesting the effect of the thicker layer but also the contributing effect of each layer with a dominance of SPUAA properties.

Figure 4d depicts the suture retention strength of the single-layer and three-layer polyurethane vascular grafts. From here it is seen that the highest suture strength was achieved by the TVG (1123 ± 144 g), followed by single-layer SPUAA (616 ± 97 g), with the lowest being those exhibited by single-layer SPUUK (396 ± 46 g) and the single-layer polyurethane blend (404 ± 57 g). These values are lower than those reported for Dacron but superior in the case of TVG to those reported for Goretex^®^ using a 2-0 catgut suture [57]. A summary of the tensile mechanical properties is reported in Table 1.

The above results show that the TVG exhibited better mechanical properties (longitudinal and circumferential strength) than MVG and single-layer vascular grafts due to its higher thickness. The TVG’s thickness was chosen because of the reported coronary artery wall thickness (0.500 mm) and because some PTFE grafts reach up to 0.650 mm, while Dacron grafts are reported to have a wall thickness of 0.33 mm. Considering the complex stress state of hyperelastic rings (tension, bending and shear forces), it was not possible to establish equivalent stresses for rings of different thicknesses. Nevertheless, analytical solutions exist to address the difference in thickness for circumferential tension, such as treating the rings as curved beams. However, this approach was not pursued for the current paper. It is important to note that standard 7198 only considers the structural and geometrical properties, such as force and diameter, rather than intrinsic properties tied to the vascular graft wall thickness, i.e., only those properties with commercial or real-world relevance are considered. However, in the case of longitudinal tension, it can be normalized by its corresponding area, assuming that there is no compression at the grips, that the testing area is in the middle or far from the grips where the shape is retained and that there is no misalignment. Hence, the outcomes can be standardized based on their thickness or area. Due to this standardization, during longitudinal testing, MVG and SPUAA experienced higher stress as compared to TVG, possibly due to the creation of a single bulk system (miscible or immiscible) instead of an anisotropic layered structure, indicating a combined contribution of various polyurethanes.

A drawback of our research pertains to the absence of assessment concerning the viscoelastic characteristics of the polyurethanes utilized in creating the vascular grafts. These properties are known to be time-dependent and were not measured due to the lack of a suggested method by the ISO 7198 standard. However, a review of the literature indicated that stress relaxation and viscoelastic recovery are meaningful, as demonstrated by Amabili et al. [58], who underscored the significance of cyclic axisymmetric diameter alterations acquired under physiological pulsatile conditions. Studies have shown that Dacron grafts are noticeably stiffer around their circumference than the aortic vessels they replace, resulting in a significant mechanical mismatch between the grafts and the native tissue. While we did not perform dynamic experiments, our circumferential and longitudinal quasi-static testing observations aligned with the ISO 7198 standard, indicating that longitudinal deformation is more significant than circumferential deformation, with no distinct J-shape observed. Additionally, the slopes (N/mm) at any deformation were higher in the circumferential direction than in the longitudinal direction, demonstrating the anisotropic stiffness of our polyurethanes and supporting the results of prior dynamic experiments.

### 3.3. Burst Strenght

**Figure 5:**
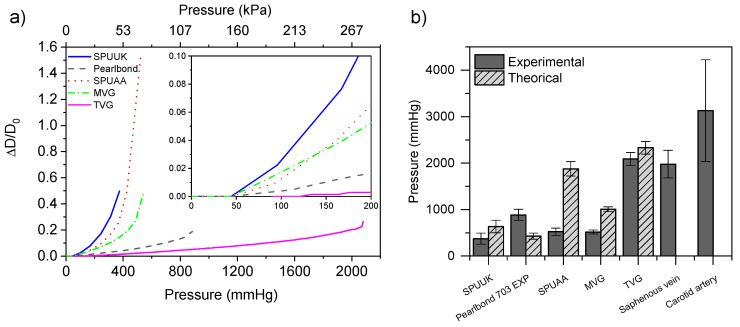
(**a**) Change in external diameter (circumferential strain) as a function of internal pressure for SVG, MVG and TVG and (**b**) experimental and theoretical burst pressure estimated via a ring tensile test using the failure diameter and those reported for human carotid arteries and human saphenous veins.

The changes in the circumferential strain with respect to the internal pressure of the vascular grafts can be seen in Figure 5a. From this figure, it is evident that single-layer SPUUK grafts experienced a high change in diameter (circumferential strain) under pressure while exhibiting the lowest burst pressure (373 ± 120 mmHg) compared to the other grafts with the same wall thickness. In contrast, single-layer SPUAA grafts showed the maximum circumferential strain ($\varepsilon_{\theta }=$ 151.5 ± 49.4%) of all grafts but needed more pressure to reach the same strain as SPUUK. The Pearlbond 703 EXP graft showed the lowest change in diameter under pressure, i.e., it was the least compliant and most stiff. The measured burst pressure of Pearlbond 703 EXP exhibited the highest value (884 ± 120 mmHg), which is about twice as high as that measured for the SPUUK (323 ± 120 mmHg) and SPUAA (517 ± 43 mmHg). The vascular grafts prepared via polymer blending (MVG) showed a burst pressure located between SPUUK/SPUAA and Pearlbond 703 EXP, with a value of 523 ± 78 mm Hg, which is more than four times the upper physiological pressure of 120 mm Hg and more than twice the hypertensive crisis pressure (180–220 mm Hg) [27]. In summary, the burst strength of single-layer grafts had the following order: $P_{BS}^{SPUUK}<P_{BS}^{SPUAA}<P_{BS}^{M.V.G.}<P_{BS}^{Pearlbond}$. Finally, the burst strength of the three-layer vascular graft (2087 ± 139 mmHg) with a wall thickness mimicking a coronary artery (~0.50 mm) was higher than the other types of vascular grafts. This value is much higher than any possible physiological blood pressure but consistent with saphenous veins and carotid arteries, which have burst pressures between 1680 and 2273 mmHg and between 2031 and 4225 mmHg [57,59], respectively.

**Figure 6:**
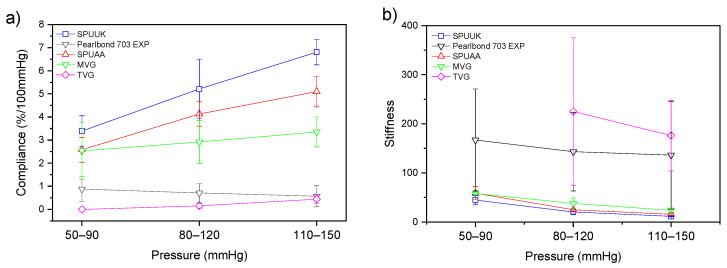
(**a**) Compliance variation with pressure (50 to 150 mmHg) for single (SPUUK, Pearlbond 703 EXP, SPUAA, MVG) and three-layer vascular grafts (TVG). (**b**) Stiffness values obtained with Equation (6).

The burst pressures measured during this study and those estimated according to the failure diameter (Equation (1)) are shown in Figure 5b. In some cases, burst strength is overestimated, while in other cases it is underestimated, suggesting a poor correlation with the experimental results. Figure 6a depicts the compliance values of the five types of vascular prostheses at different pressure levels: low (50–90 mmHg), medium (80–120 mmHg) and high (110–150 mmHg). The SPUUK, SPUAA, MVG and TVG samples showed higher compliance at high pressure levels and lower compliance at low pressure levels, indicating that compliance increases with increasing pressure. In contrast, the Pearlbond 703 EXP graft exhibited its highest compliance at a low pressure and its lowest compliance at a high pressure, indicating a decrement in compliance with increasing pressure.

The SPUUK and SPUAA grafts exhibited the highest compliance, measured at 80–120 mmHg of 5.2 ± 1.3 and 4.1 ± 0.5%/100 mmHg, respectively. This finding is noteworthy because it is closer to the physiological value for human saphenous veins, with a compliance of 4.4 ± 0.4%/100 mmHg [60]. The MVG graft showed lower compliance than SPUUK and SPUAA but a higher compliance than Pearlbond 703 EXP. Figure 6b illustrates a comparison of the stiffness measured at different pressure ranges. Consistent with the compliance values, the stiffness index was higher for TVG, indicating that the lower the compliance, the higher the stiffness index. Table 2 summarizes the burst strength and compliance values of the various polyurethane vascular grafts.

### 3.4. Fracture Surfaces of Vascular Grafts after Mechanical Testing

**Figure 7:**
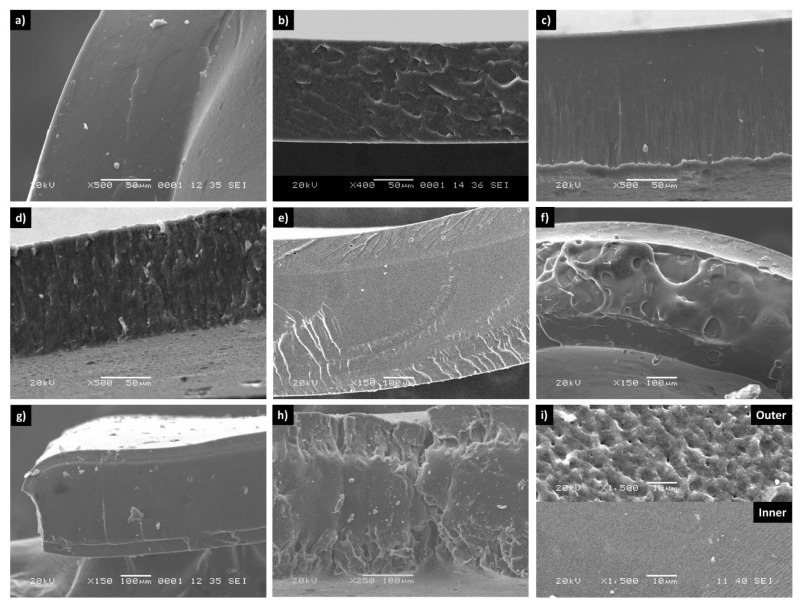
SEM micrographs of the cross-section of single-layer vascular grafts made from (**a**) SPUUK, (**b**) Pearlbond 703 EXP, (**c**) SPUAA and (**d**) MVG. (**e**) Three-layer vascular graft prepared with SPUUK, Pearlbond 703 EXP and SPUAA. (**f**) Cross-section of TVG after longitudinal testing, (**g**) circumferential testing and (**h**) the burst strength test. (**i-Inner**) The inner surface of the TVG based on SPUUK and (**i-Outer**) the outer surface based on SPUAA.

Figure 7a–e present representative SEM cross-sections of the liquid-nitrogen-fractured surfaces of SVG (SPUUK, Pearlbond 703 EXP, SPUAA and MVG) and TVG obtained via roto-evaporation. For TVG, good adhesion between the three different polyurethanes that compose the graft was observed, with a clear demarcation of the inner, middle and outer layers, (SPUUK, Pearlbond 703 EXP and SPUAA). The inner surface (Figure 7i-Inner) of the TVG based on SPUUK showed a smooth surface where the polymer was highly compacted in contact with the glass rod, while the outer surface (Figure 7i-Outer) of the TVG based on SPUAA showed a rougher surface caused by the evaporation of the solvent during the rotation. In addition, Figure 7f–h show the three-layer vascular graft after the longitudinal, circumferential and burst test, respectively. After longitudinal or circumferential failure, no delamination of the polyurethanes composing the graft was observed. However, the different compositions can be identified according to the different types of fracture. For example, after longitudinal tensile tests, the TVG showed a ductile failure in the middle thicker layer composed of Pearlbond 703 EXP, while brittle failure was observed after circumferential tensile tests. Figure 7h depicts the surface of the vascular graft after the burst strength test. The surface exhibited various fracture patterns depending on the layer composition. Specifically, the outer and inner layers, composed of SPUAA and SPUUK, respectively, displayed rougher surfaces (indicative of a more brittle fracture). In contrast, the middle layer, consisting of a commercial polyurethane called Pearlbond 703 EXP, exhibited a smoother surface (suggesting a less brittle fracture).

### 3.5. Biological Performance

#### 3.5.1. MDA-MB-231 Cytocompatibility

**Figure 8:**
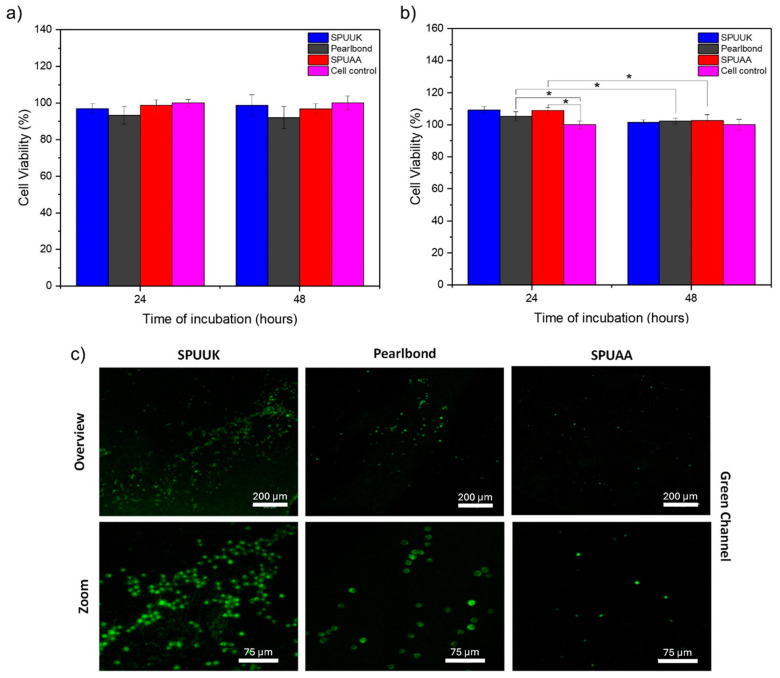
Viability of MDA-MB-231 cells upon exposure to extracts from the different polyurethanes. (**a**) CCK-8 and (**b**) crystal violet assay. (*) The values shown are the mean ± SD. A value of *p*-value < 0.05 was considered significant. (**c**) Confocal fluorescence micrographs of fibroblast L–929 cells 24 h after seeding onto selected films. Live cells (stained with calcein-AM) appear in green.

Figure 8a shows the viability of MDA-MB-231 cells determined by means of the CCK-8 assay after 24 and 48 h of exposure to extracts of the different polyurethanes. Metabolically active cells can reduce the WST-8 tetrazolium salt present in CCK-8 into a water-soluble formazan product. Nonviable cells rapidly lose their ability to reduce the WST-8. Therefore, the production of the colored formazan products is proportional to the number of viable cells [61]. The three layers comprising the TVG exhibited values ranging from 90% to 100%, surpassing the minimum threshold of 70% required for biomaterials to be considered cytocompatible [49]. Figure 8b shows the viability of MDA-MB-231 cells obtained using the crystal violet assay to assess cell membrane integrity, upon exposure to extracts of the different polyurethanes, normalized with respect to the control (100%) cells at 24 and 48 h. All samples exhibited cell viability above the 70% required, with values around 100%.

#### 3.5.2. Cell Adhesion and Proliferation

The cytocompatibility of the polyurethane films was also evaluated via the well-known calcein AM/propidium iodide assay (Live/Dead). Calcein-AM is a small molecule dye that fluoresces when cleaved by esterases in viable cells, staining them in green [62], whereas propidium iodide is a fluorescent molecule that binds preferentially to DNA, staining membrane-disrupted cells in red. As such, the staining by these two dyes proves useful to discriminate between populations of live and dead cells [61]. The results from the live/dead assays are shown in Figure 8c, where few fibroblast cells appeared on the surface with little proliferation of Pearlbond 703 EXP and SPUAA.

Even when phalloidin staining was not performed to assess proper cell morphology, SPUUK exhibited good fibroblast adhesion. In terms of the water contact angle, there was no difference between the three polyurethanes (87.9 ± 3.3°, 84 ± 2.8° and 87.3 ± 1.4° for SPUUK, Pearlbond 703 EXP and SPUAA, respectively). Still, in terms of surface roughness, there was a slight difference (Ra = 1.02 ± 0.3 µm, 0.52 ± 0.09 µm and 0.31 ± 0.11 µm for SPUUK, Pearlbond 703 EXP and SPUAA, respectively) that can explain the observed outcome. As demonstrated by the screening tests of cytocompatibility presented in the previous section, the moderate adhesion and proliferation of fibroblast cells onto Pearlbond 703 EXP and SPUAA are not expected to be related to any toxic effect of these materials. Instead, they could be related to their surface’s structural parameters, such as the topography, elasticity and water contact angle.

Taking all of these results together, it was observed that both cell metabolic activity and membrane integrity were uncompromised in the presence of all polyurethane extracts, even when solvents (DMF), monomers (isocyanates) or catalysts (tin (II) 2-ethylhexanoate) were used during their synthesis, probably due to their low concentration and exposure time. In contrast, the direct cytotoxicity test showed that only SPUUK could support fibroblast adhesion due to a slightly rougher surface. In this regard, many polymers are not cytotoxic but show poor or little cell adhesion. However, it must be noted that cell adhesion is mainly mediated by protein adsorption on the surface and not only by chemical composition, hydrophilic/hydrophobic balance, surface roughness, etc. Therefore, the extra lysine amino groups in SPUUK promoted the interaction with a negatively charged cell membrane.

#### 3.5.3. Hemocompatibility

**Figure 9:**
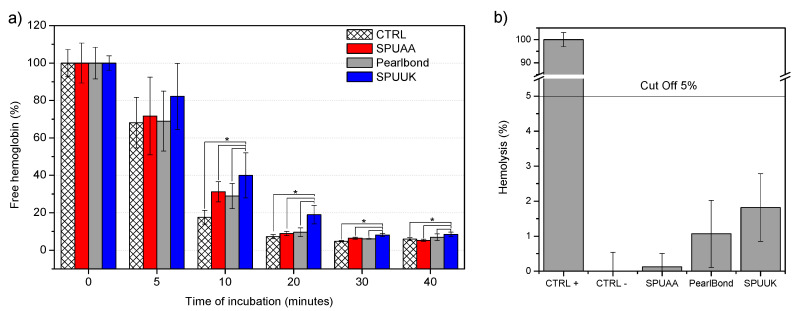
(**a**) Clotting time study on polyurethane films. A *p*-value < 0.05 was considered significant (*) and was found at 10, 20, 30 and 40 min between SPUUK and the other conditions. The results are normalized to the plastic control. The values shown are the mean ± SD. Experiments were repeated three times for three different donors, with n = 3 each time. (**b**) Hemolysis percentage of different polyurethanes.

Figure 9a illustrates the progression of thrombus formation over time, with observations made at 5, 10, 20, 30 and 40 min of contact between whole blood and the various polyurethane surfaces. At 5 min, no significant difference between surfaces was noted, and the thrombus barely started to form. However, after 10 min, the amount of free hemoglobin was significantly higher in the SPUUK polyurethane than in the other polyurethanes for all incubation times evaluated. This is advantageous for the TVG because the inner layer, which will be in direct contact with blood, comprises SPUUK. Once free hemoglobin values reach 25%, clot formation becomes evident. According to studies by Boccafoschi et al. [63], Teflon^®^ shows values of 25% at 20 min. In our research, SPUUK exhibited values of approximately 25% at around 15 min, whereas Pearlbond 703 EXP and SPUAA exhibited equivalent values at around 10 min. The blood compatibility of polyurethanes was also evaluated by measuring the cell hemolysis percentage during contact with red blood cells in vitro [64]. The percentage of hemolysis depicts the degree of destruction of erythrocytes when they meet the polyurethane films. Therefore, the higher the number of broken erythrocytes, the higher the value of the hemolysis percentage is. A higher degree of hemolysis indicates poor hemocompatibility of the biomaterial. Figure 9b illustrates the hemolysis rate values of different polyurethane films. The degree of hemolysis in the presence of SPUAA was 0.12 ± 0.38%, for Pearlbond 703 EXP it was 1.07 ± 0.96% and for SPUUK it was 1.82 ± 0.96%. However, the hemolysis values of all three kinds of materials were under the permissible limit of 5% [65].

## 4. Conclusions

A three-layered polyurethane vascular graft that mimics the thickness of each tunica in an artery was developed through the roto-evaporation method. The properties of these vascular grafts were assessed using the ISO 7198 standard. It was found that the 500 µm-wall-thickness TVG made from SPUUK (tunica intima), Pearlbond 703 EXP (tunica media) and SPUAA (tunica adventitia) exhibited the highest circumferential tensile strength (7.17 ± 0.37 N/mm) and longitudinal forces (101.3 ± 4.0 N) when compared to single-layer vascular grafts (100 µm wall thickness) made from the same polyurethanes. In addition, the three-layer vascular graft showed the highest suture strength (11.0 ± 1.4 N) and exhibited higher burst strength values (2087 ± 139 mmHg) than native vessels. Lower compliances (0.15 ± 0.1 and 0.44 ± 0.18 at 80–120 mmHg and 110–150 mmHg, respectively) and higher stiffness indexes (4054.7 ± 0.01) were achieved for TVG. Although the three polyurethane extracts exhibited non-cytotoxic behavior when cultured directly on the surface, they showed poor fibroblast adhesion, except for the lysine-based polyurethane (SPUUK). In agreement with this, the inner layer of the TVG, a lysine-based polyurethane, exhibited 80% free hemoglobin after 5 min (longer than the plastic control), which was reduced to 10% after 40 min, indicating a long clotting time. Additionally, the inner layer showed low hemolysis (1.82 ± 0.96%), which is below the acceptable 5% threshold for this parameter.

Determining mechanical properties is a suitable way of assessing the performance of a vascular graft, with higher only sometimes being better. The tensile mechanical test of SPUUK and SPUAA films showed that they have higher strength and deformation than Pearlbond 703 EXP but a lower modulus. Therefore, the three-layer structure improves the grafts’ tensile properties and allows them to perform better than the corresponding immiscible polymer blend. However, this led to compliances that were lower than most natural tissues.

Based on these results, it is concluded that for achieving higher compliances or compliances akin to those of native tissues, the thickness and composition of the media layer (the thicker one) are the most important factors in the development of synthetic grafts. Therefore, a three-layer synthetic vascular graft must be at least 50–100 µm thick for high compliance when the segmented polyurethanes used as a media layer (Pearlbond 703 EXP in this study) exhibit a high Young’s modulus. Alternatively, it is suggested that compliances can be increased in a tubular form by increasing porosity. Finally, the polyurethane used for the media layer can be replaced with a low-modulus polyurethane (with no phase separation) for higher compliance.

In conclusion, the roto-evaporation method is a good alternative for preparing layered tubular structures as it allows for different material compositions and thicknesses in a shorter period. However, further studies are needed to evaluate these synthetic grafts’ long-term performance and biocompatibility before they can be used in clinical applications. To develop a final vascular substitute, it is recommended to include future studies related to their performance in animal models, their viscoelastic properties and more hemocompatibility tests that include platelet or protein adhesion and to observe it via SEM.

## Figures and Tables

**Table 1:** Tensile mechanical properties of films and tubular grafts made from single-layer SPUUK, Pearlbond 703 EXP, SPUAA, the mixture vascular graft and the three-layer vascular graft ^a^.

	SPUUK	Pearlbond 703 EXP	SPUAA	MVG	TVG ^b^
	**Film**				
$E_{10\%}$ **[MPa]**	3.4 ± 0.4	17.9 ± 3.3	3.3 ± 0.2	10.6 ± 0.3	9.3 ± 2.4
$\sigma_{max}$ **[MPa]**	12.76 ± 1.42	7.04 ± 0.73	30.77 ± 4.11	6.8 ± 0.4	9.4 ± 1.6
$\varepsilon_{max}$ **[%]**	1330 ± 125	521 ± 101	1430 ± 88	650 ± 83	930 ± 242
$F_{max}$ **[N]**	14.5 ± 2.5	3.5 ± 0.5	18.8 ± 2.3	4.0 ± 1.4	13.0 ± 3.1
$\delta_{max}$ **[mm]**	158 ± 14	59 ± 14	182 ± 17	65 ± 14	120 ± 31
	**Graft (longitudinal)**				
$F_{L}^{max}$ **[N]**	26.1 ± 6.5	18.1 ± 4.3	49.8 ± 8.3	40.8 ± 5.2	101.3 ± 4.0
$\delta_{L}^{max}$ **[mm]**	404 ± 40	307 ± 86	373 ± 18	443 ± 26	484 ± 29
	**Graft (circumferential)**				
**wall thickness [mm]**	0.130 ± 0.034	0.130 ± 0.019	0.130 ± 0.027	0.140 ± 0.01	0.499 ± 0.021
$F_{C}^{max}/2L_{C}$ **[N/mm]**	1.49 ± 0.39	0.87 ± 0.11	5.00 ± 0.61	2.70 ± 0.20	7.17 ± 0.37
$F_{C}^{max}$ **[N]**	19.2 ± 4.9	11.3 ± 1.4	68.7 ± 8.5	37.3 ± 3.4	107.5 ± 6.1
$\delta_{C}^{max}$ **[mm]**	48.7 ± 4.5	42.2 ± 7.6	56.2 ± 2.8	56.9 ± 2.2	65.6 ± 1.8
**‡**$\;\sigma_{\theta }^{max}$ **[MPa]**	11.55 ± 1.48	6.76 ± 0.88	40.10 ± 6.24	18.97 ± 1.49	14.49 ± 0.71
	**Suture retention strength**				
F **[N]**	3.9 ± 0.5	5.3 ± 1.1	6.0 ± 1.0	4.0 ± 0.6	11.0 ± 1.4
***F*** **[g]**	396 ± 46	544 ± 111	616 ± 97	404 ± 57	1123 ± 144

^a^ Obtained according to ISO 7198 standard. ^b^ Coronary artery-like wall thickness ~0.50 mm. **^‡^** Calculated using Equation (2).

**Table 2:** Burst strength and compliances of vascular grafts based on single-layer SPUUK, Pearlbond 703 EXP, SPUAA, the mixture blend vascular graft and the three-layer vascular graft ^a^.

	SPUUK	Pearlbond 703 EXP	SPUAA	MVG	TVG ^b^
**Wall thickness [mm]**	0.132 ± 0.008	0.130 ± 0.012	0.142 ± 0.010	0.140 ± 0.004	0.500 ± 0.010
**External diameter [mm]**	6.27 ± 0.02	6.26 ± 0.02	6.29 ± 0.03	6.28 ± 0.01	7.01 ± 0.01
**Burst pressure [mmHg]**	373 ± 120	884 ± 120	517 ± 43	523 ± 78	2087 ± 139
$P_{i}^{BS}$ **[mmHg] Df ^c^**	634 ± 134	428 ± 66	1877 ± 155	1005 ± 51	2330 ± 136
$P_{i}^{BS}$ **[mmHg] Du ^c^**	3642 ± 936	2126 ± 267	12,230 ± 1517	6350 ± 628	15,834 ± 1372
**Deformation [%]**	49.3 ± 8.2	18.8 ± 2.6	151.5 ± 49.4	48.2 ± 7.6	40 ± 15.3
***C* 50**–**90 [%100/mmHg]**	3.03 ± 1.06	0.88 ± 0.55	2.58 ± 0.54	2.53 ± 1.22	0.0 ± 0.0
***C* 80**–**120 [%100/mmHg]**	5.21 ± 1.29	0.71 ± 0.4	4.13 ± 0.53	2.92 ± 0.93	0.15 ± 0.1
***C* 110**–**150 [%/100 mmHg]**	6.81 ± 0.55	0.57 ± 0.46	5.1 ± 0.66	3.35 ± 0.65	0.44 ± 0.18
**Stiffness index 80**–**120 mmHg**	20.3 ± 3.9	143 ± 80	24.8 ± 3.2	37.6 ± 11.1	225 ± 150

^a^ Obtained according to ISO 7198 standard. ^b^ This graft had a wall thickness mimicking coronary artery ~0.5 mm. ^c^ Burst pressure computed with Lame’s equation for unload diameter (Du) and failure diameter (Df).

## Data Availability

The data presented in this study are available upon request from the corresponding author.
